# Case Report: Pulmonary Kaposi Sarcoma in a non-HIV patient

**DOI:** 10.12688/f1000research.7137.1

**Published:** 2015-10-07

**Authors:** Arber Kodra, Maciej Walczyszyn, Craig Grossman, Daniel Zapata, Tarak Rambhatla, Bushra Mina

**Affiliations:** 1 Department of Internal Medicine, Lenox Hill Hospital, New York, NY, 10065, USA

**Keywords:** Kaposi Sarcoma, Lymphoma, Pulmonary, Bronchoscopy

## Abstract

Kaposi Sarcoma (KS) is an angioproliferative tumor associated with human herpes virus 8 (HHV-8).  Often known as one of the acquired immunodeficiency syndrome (AIDS)-defining skin diseases, pulmonary involvement in KS has only been discussed in a handful of case reports, rarely in a non-HIV patient. Herein we report the case of a 77 year-old- male who presented with a 6-week history of progressive dyspnea on exertion accompanied by productive cough of yellow sputum and intermittent hemoptysis. His past medical history was significant for Non-Hodgkin’s Follicular B-Cell Lymphoma (NHL). Patient also had biopsy-confirmed cutaneous KS. His physical exam was notable for a 2cm firm, non-tender, mobile right submandibular lymph node.  Lungs were clear to auscultation. He had multiple violet non-tender skin lesions localized to the lower extremities. CT scan of the chest showed numerous nodular opacities and small pleural effusions in both lungs. A thoracenthesis was performed, showing sero-sanguineous exudative effusions. Histopathology failed to demonstrate malignant cells or lymphoma. A subsequent bronchoscopy revealed diffusely hyperemic, swollen mucosa of the lower airways with mucopurulent secretions. Bronchoalveolar lavage PCR for HHV-8 showed 5800 DNA copies/mL.  It was believed that his pulmonary symptoms were likely due to disseminated KS.  This case illustrates the potential for significant lung injury from KS. It also demonstrates the use of PCR for HHV-8 to diagnose KS in a bronchoalveolar lavage sample in a case when bronchoscopic biopsy was not safe. Furthermore, this case is unique in that the patient did not match the typical KS subgroups as HIV infection and other immune disorders were ruled out. Recognition of this syndrome is critical to the institution of appropriate therapy. As such, this case should be of interest to a broad readership across internal medicine including the specialties of Pulmonology and Critical Care.

## Case report

The patient was a 77 year-old Hispanic male who initially presented to the emergency room with a 6-week history of progressive dyspnea on exertion, cough productive of yellow sputum, intermittent hemoptysis, worsening fatigue and weight loss of 10 lbs. On admission, he denied fever, chills, chest pain, sick contacts, recent travel, or tuberculosis exposure and history. His past medical history was significant for Non-Hodgkin follicular B cell lymphoma (NHL) diagnosed in 1997, treated with Rituximab, Cyclophosphamide, Hydroxydaunorubicin, Oncovin and Prednisone (RCHOP) three times. Last treatment was completed 1 year prior to presentation. He also had biopsy-confirmed cutaneous Kaposi’s Sarcoma (KS), identified within the same year of presentation, treated with pegylated liposomal doxorubicin 20 mg/m ^2^ once every 21 days for two cycles.

On admission, he had a low grade fever and was tachycardic. His physical exam was remarkable for a 2 cm firm, non-tender, mobile right submandibular lymph node. Lungs were clear to auscultation bilaterally. He was grossly anasarcic with 4 (+) pitting edema of the lower extremities. He also had multiple violet, non-tender skin lesions localized to the lower extremities, mainly around the medial aspect of his ankles and anterior thighs bilaterally.

His labs revealed a normal cardiac panel, a white blood cell (WBC) count of 8,100/mm ^3^ with no leukocytosis. Manual differentiation of the white blood cells showed 28% neutrophils, 48% lymphocytes, 17% monocytes and 5% eosinophils. Urinalysis and urine culture were negative for infections. Blood cultures were positive for *Streptococcus pneumoniae.*

**Figure 1.**
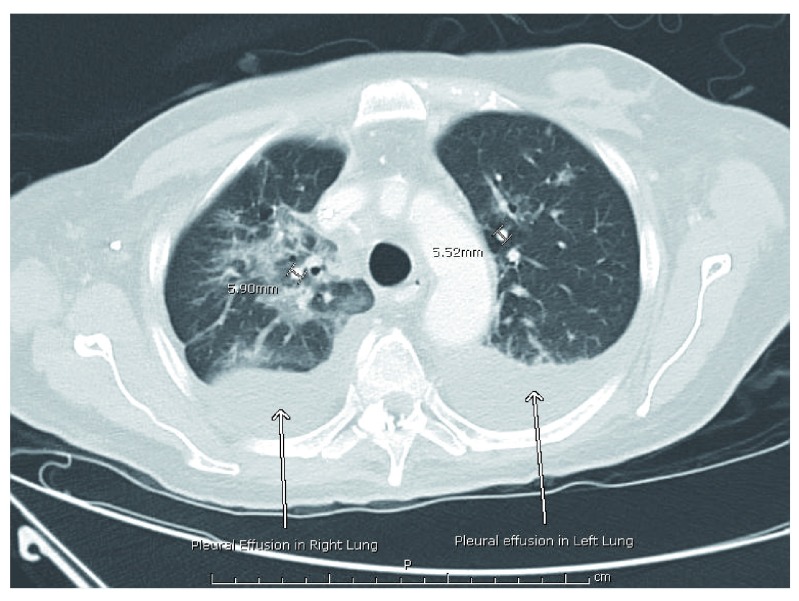
CT of the chest on admission to hospital. CT of the chest demonstrating several nodular opacities throughout both lungs. Two nodules are measured to show size. Arrows point to pleural effusions on both lungs.

A chest X-ray done on admission showed a right lung consolidation consistent with pneumonia thus the patient was started on empiric vancomycin 1gm every 12 hours and piperacillin-tazobactam 4.5gm every 8 hours for a duration of 8 days. A computerized axial tomographic (CT) scan of the chest, abdomen and pelvis, showed numerous nodular opacities throughout each lung which were suspected to be consistent with KS as well as small pleural effusions in both lungs ( Figure 1).

**Figure 2.**
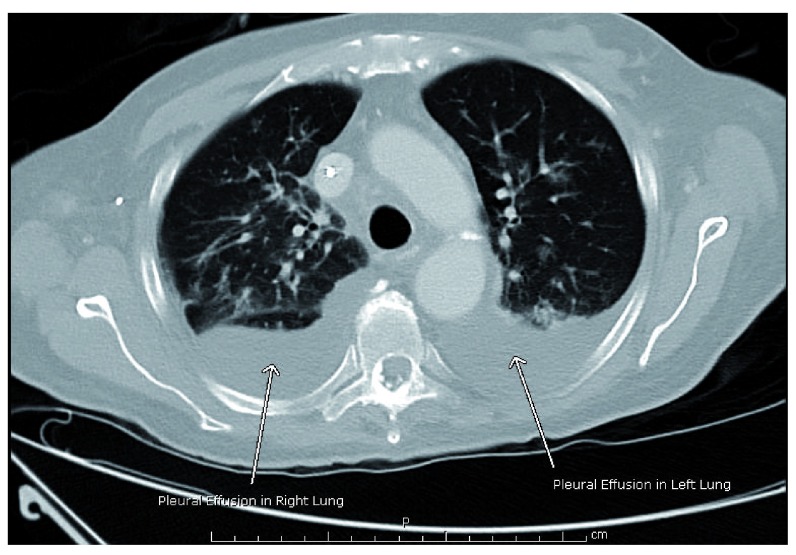
CT of the chest during hospitalization. CT of the chest showing nodular opacities that persisted in both lung fields and worsening bilateral pleural effusions (arrows).

Despite completing a course of antibiotics for pneumonia, his symptoms did not improve. A follow-up CT scan of the chest demonstrated that the bilateral pleural effusions had worsened ( Figure 2). Patient underwent a thoracentesis of the larger effusion in the right lung which showed a sero-sanguineous and exudative fluid based on chemistry analysis. Histopathology failed to demonstrate malignant cells.

**Figure 3.**
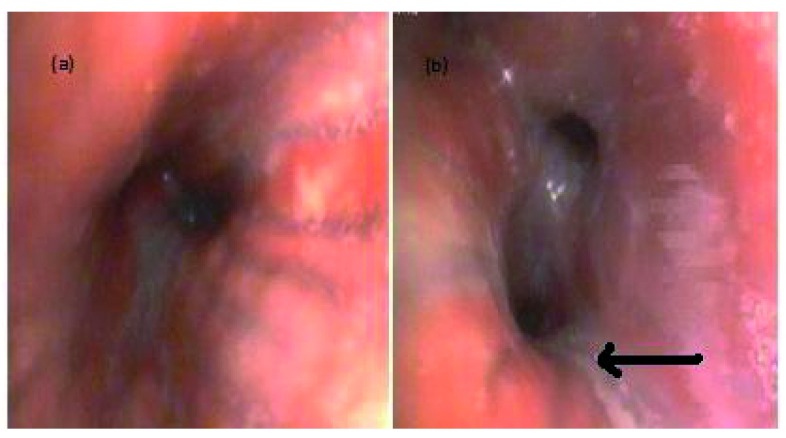
Bronchoscopy performed during hospitalization. Photographs of the bronchoscopy performed after patient’s symptoms were not improving with appropriate antibiotic therapy. ( **a**) Diffusely hyperemic and edematous mucosa of lower airways. ( **b**) Arrow points to airway with significant mucopurulent secretions.

A bronchoscopy was also performed, revealing diffusely hyperemic and edematous mucosa of the lower airways with mucopurulent secretions ( Figure 3). Biopsy was not performed at this time given concern for endobronchial bleeding. Bronchoalveolar lavage (BAL) stains and cultures were negative for bacteria, fungi or acid fast bacilli. *Pneumocystis jiroveci* PCR and fluid cytology were also negative but PCR for HHV-8 showed 5800 DNA copies/mL. The patient had a HIV p24 rapid antigen test done which was negative.

After two weeks of supportive treatment, the patient’s symptoms began to improve. He continued to have numerous indurated confluent lesions on his body and face consistent with KS. His pulmonary symptoms were thought to be due to disseminated KS. Following significant discussions with the patient and his family regarding the poor prognosis of his disease, he decided not to undergo any further antineoplastic treatments and eventually was discharged home with hospice.

## Discussion of diagnosis

Kaposi Sarcoma (KS) is an angioproliferative tumor associated with human herpes virus 8 (HHV-8) ^1, 2^. It is one of the AIDS-defining skin diseases and is strongly linked to male homosexual behavior ^3, 4^. Four clinical variants of KS have been described: classic, African, iatrogenic and AIDS-related ^5– 10^. The classic variant mainly affects elderly men of Eastern European Jewish and Mediterranean origin ^7– 9^. The African, or endemic type, affects primarily men in the 4 ^th^ decade of life in East and Central Africa ^10^. Its clinical presentation is similar to the classic form but with a more aggressive variant that responds poorly to conventional treatment ^10^. The third variant, iatrogenic KS, is related to chronic immunosuppressive drugs used in organ-transplant recipients or cancer patients ^5– 10^. This variant tends to be more aggressive, involving lymph nodes, mucosa and visceral organs. AIDS-related KS is an aggressive epidemic form that commonly affects patients with immunosuppression from AIDS ^3– 5^.

Pulmonary involvement in KS has only been discussed in a handful of case reports, very few of which were in non-HIV patients ^11– 16^. This is likely due to the lack of published evidence of KS in general, as well as the presence of multiple co-morbidities in most patients which may mask clear identification of pulmonary KS. In most cases, it occurs in conjunction with more extensive muco-cutaneous disease ^12– 16^. Unique manifestations that distinguish KS from other pathologic processes in the lungs have not been identified. The most common characteristics of pulmonary KS include peri-broncho-vascular and nodular opacities, thickened interlobular septa and pleural effusions ^17– 21^.

Signs and symptoms such as shortness of breath, hypoxemia, and dry cough are common in pulmonary KS. Hemoptysis, fever, chest pain and respiratory failure can also occur. Additionally, enlarged mediastinal lymph nodes are frequently seen in patients with this disease ^16– 21^.

The diagnosis of pulmonary involvement in KS can often be made by a combination of clinical, radiographic and laboratory findings, in conjunction with results of a transbronchial biopsy ^21, 22^. Radiographic findings in pulmonary KS are varied and include segregated pulmonary nodules, pleural effusions, and hilar or mediastinal lymphadenopathy, all of which were also evident in our patient ^23– 25^. Several polymerase chain reaction (PCR) assays employing primers unique for HHV-8 have been described ^26, 27^. HHV-8 DNA can be identified using PCR in biopsies of KS, including AIDS-associated KS, classic KS, and endemic KS. Studies have also shown that HHV-8 DNA can be detected in the BAL of patients with pulmonary KS, as was evident in our case ^28– 30^.

Our patient did not match the typical subgroups as HIV infection and other immune disorders were ruled out. Furthermore, it has been shown that KS, in non-HIV patients, clinically resembles classic KS but occurs at a younger age, is limited to the skin, and is associated with a good prognosis ^5^. Our patient, however, demonstrated both dermatologic and pulmonary manifestations suggesting a disseminated and aggressive form of the disease.

## Treatment and management

The published literature on the treatment of KS consists mostly of retrospective series and case reports ^31– 34^. At the time of this case report, we are aware of only a few prospectively randomized trials to date that compare different treatments for KS, most of which were for AIDS-related KS ^35– 37^. This is likely due to the lack of published evidence of the disease and the presence of co-morbidities in most patients, which may limit treatment options such as in our case.

Currently, antiretroviral therapy (ART) is the first-line therapy for pulmonary KS as it is often seen in patients with HIV/AIDS ^31– 33^. The first-line treatment for KS in patients with CD4 counts greater than 350 cells/μL is still unclear and treatment has generally been palliative in nature.

Only systemic treatments, including chemotherapy and immunomodulators, have shown potential to cause regression in all sites of disease ^36– 38^. These include pegylated liposomal doxorubicin, vinblastine, alone or in combination with bleomycin, paclitaxel, oral etoposide, vinorelbine, gemcitabine and the immunomodulator recombinant interferon alfa (IFNa). Overall response rates for all of these therapies have been reported to be high and the treatments are generally well tolerated, even in the elderly population. Only one randomized trial has been conducted in which two different systemic therapies, etoposide and vinblastine, were compared in non-AIDS related KS ^35^. That study showed no significant differences between the two treatments with regard to response rate or survival.

Despite the lack of randomized trials demonstrating superiority, most clinicians consider pegylated liposomal doxorubicin the first-line therapy of choice based on a retrospective multicenter series of patients with classic KS without evidence of HIV which showed ≥50% decrease in the number of measurable lesions and the absence of new cutaneous lesions for at least eight weeks in 71% of treated patients ^38^.

Our patient received treatment with liposomal doxorubicin prior to admission resulting in improvement of his cutaneous lesions. Doxorubicin was planned to be started prior to discharge, but the patient declined further chemotherapy, electing to establish hospice care.

Radiotherapy is also an accepted treatment for all forms of KS. However, due to the tendency of new lesions to develop as well as the persistence of HHV-8, despite improvement of local lesions and symptoms, there is no consensus as to when to choose radiotherapy over systemic therapy ^39, 40^.

## Conclusion

KS in a non-immunocompromised patient is an infrequent occurrence and pulmonary involvement makes the diagnosis even more difficult as only a handful of cases in this patient population are present in the literature. Traditional measures of treatment are aimed at curbing the underlying immunosuppression, making it difficult to treat in individuals with normal immune function. Pulmonary involvement can be ascertained by a combination of clinical, radiographic and laboratory findings, in conjunction with results of a transbronchial biopsy.
