# Supplementary material for: Case Report: Pulmonary Kaposi Sarcoma in a non-HIV patient
Source: F1000Res. 2015 Oct 7;4:1013. [Version 1] doi: 10.12688/f1000research.7137.1 (PMC4654435; doi:10.12688/f1000research.7137.1)
Supplement: Peer review history

# Peer review: Referee response for version 1

**DOI:** 10.5256/f1000research.7687.r11204

**Published:** 2015-11-12

**Authors:** Chunxue Bai

**Affiliations:** 1 Department of Pulmonary Medicine, Fudan University School of Medicine, Zhongshan Hospital, Shanghai, China

Arber Kodra *et al. *presented a case of KS that involved the pulmonary in a HIV negative patient. This patient has past medical history of NHL and cutaneous KS. PCR for HHV-8 in BALF showed 5800 DNA copies/ ml and bronchoscopy showed diffusely hyperemic and edematous mucosa of lower airways. The authors claimed that this patient’s pulmonary symptoms were likely due to disseminated KS and the useful of PCR for HHV-8 to diagnose KS in a BALF sample in a case when bronchiscopic biopsy was not safe.

1. KS is thought to originate from HHV8 infected lymphatic endothelial cells. HHV8 is recognized as an essential and necessary factor in the pathogenesis of KS. However, not all HHV-8 infected individuals will develop the disease. The authors examined PCR for HHV-8 in BALF sample; however, it will be more convincing if the author could provide pathological results to demonstrate the patient pulmonary symptoms were likely due to disseminated KS.
2. In this case, the past medical history of this patient was significant for Non-Hodgkin’s Follicular B-Cell Lymphoma. NHL may also lead to nodular opacities throughout both lungs.
3. The abnormal cells of KS form purple, red, or brown patches, plaques or tumors on the skin. It will be better if the authors could provide some pictures of cutaneous lesions.
4. It is a little puzzled that the “lung were clear to auscultation.”, whereas CT scan showed pleural effusions on both lungs.

I have read this submission. I believe that I have an appropriate level of expertise to confirm that it is of an acceptable scientific standard.

# Peer review: Referee response for version 1

**DOI:** 10.5256/f1000research.7687.r10719

**Published:** 2015-11-09

**Authors:** Michael O'Connor

**Affiliations:** 1 Department of Anesthesia and Critical Care, University of Chicago, Chicago, IL, USA

This very nice case report serves to remind practitioners everywhere the Kaposi's sarcoma both occurs independently of HIV disease, and can affect organ systems other than the skin. Modern diagnostic techniques and imaging, used as described in this report, allow us to document manifestations of Kaposi's that were previously undocumented.

I have read this submission. I believe that I have an appropriate level of expertise to confirm that it is of an acceptable scientific standard.
